# Comparative Evaluation of the Phytochemical Profiles and Antioxidant Potentials of Olive Leaves from 32 Cultivars Grown in China

**DOI:** 10.3390/molecules27041292

**Published:** 2022-02-15

**Authors:** Chengcheng Zhang, Xiaoting Xin, Jianming Zhang, Shenlong Zhu, Erli Niu, Zhongjing Zhou, Daqun Liu

**Affiliations:** 1 Food Science Institute, Zhejiang Academy of Agricultural Sciences, Hangzhou 310021, China; zccwsf@126.com (C.Z.); xxt4228@sina.cn (X.X.); zhangjianming@zaas.ac.cn (J.Z.); 2 Institute of Crop and Nuclear Technology Utilization, Zhejiang Academy of Agricultural Sciences, Hangzhou 310021, China; zhusl@zaas.ac.cn (S.Z.); niuerli@zaas.ac.cn (E.N.); 3 State Key Laboratory for Managing Biotic and Chemical Threats to the Quality and Safety of Agro-Products, Zhejiang Academy of Agricultural Sciences, Hangzhou 310021, China; zhouzj@zaas.ac.cn

**Keywords:** olive leaves, cultivars, phenolic compounds, flavonoids, secoiridoids, antioxidants

## Abstract

Olives (*Olea europaea* L.) are a significant part of the agroindustry in China. Olive leaves, the most abundant by-products of the olive and olive oil industry, contain bioactive compounds that are beneficial to human health. The purpose of this study was to evaluate the phytochemical profiles and antioxidant capacities of olive leaves from 32 cultivars grown in China. A total of 32 phytochemical compounds were identified using high-performance liquid chromatography–electrospray ionization–tandem mass spectrometry, including 17 flavonoids, five iridoids, two hydroxycinnamic acids, six triterpenic acids, one simple phenol, and one coumarin. Specifically, olive leaves were found to be excellent sources of flavonoids (4.92–18.29 mg/g dw), iridoids (5.75–33.73 mg/g dw), and triterpenic acids (15.72–35.75 mg/g dw), and considerable variations in phytochemical content were detected among the different cultivars. All tested cultivars were classified into three categories according to their oil contents for further comparative phytochemicals assessment. Principal component analysis indicated that the investigated olive cultivars could be distinguished based upon their phytochemical profiles and antioxidant capacities. The olive leaves obtained from the low-oil-content (<16%) cultivars exhibited higher levels of glycosylated flavonoids and iridoids, while those obtained from high-oil-content (>20%) cultivars contained mainly triterpenic acids in their compositions. Correspondingly, the low-oil-content cultivars (OL3, Frantoio selection and OL14, Huaou 5) exhibited the highest ABTS antioxidant activities (758.01 ± 16.54 and 710.64 ± 14.58 mg TE/g dw, respectively), and OL9 (*Olea europaea* subsp. Cuspidata isolate Yunnan) and OL3 exhibited the highest ferric reducing/antioxidant power assay values (1228.29 ± 23.95 mg TE/g dw and 1099.99 ± 14.30 mg TE/g dw, respectively). The results from this study may be beneficial to the comprehensive evaluation and utilization of bioactive compounds in olive leaves.

## 1. Introduction

The olive tree (*Olea europaea* L.) is a famous woody oil species native to the Mediterranean and is one of the most important industrial crops in the world [1]. To meet the high demand for olive oil and table olives, which represent olive products with health benefits, olive trees have been cultivated around the world, including in South America, India, Australia, and China [2]. Given their wide distribution, olive trees generate large quantities of by-products, which can cause substantial environmental issues. Olive leaves are the most abundant by-products and are generated from the pruning of olive trees, harvesting of olive fruits, and olive oil processing. Previously, the leaves were typically used as animal feed or incinerated. These applications have no commercial value and can cause resource waste and environmental damage [3]. Olive leaves possess numerous properties beneficial to human health, due to their high content of bioactive compounds. Reports have shown that olive leaves are rich in a wide variety of bioactive compounds, including flavonoids (luteolin-7-O-glucoside, rutin, apigenin, and luteolin), phenolic acids (caffeic acid, ferulic acid), secoiridoids (oleuropein, verbascoside), and pentacyclic triterpenes [1,4]. All of these compounds have remarkable biological properties, including antimicrobial, antiviral, anti-inflammatory, hypoglycemic, and antioxidant activities [5,6]. As a result, the utilization of olive leaves to obtain value-added products rich in bioactive compounds could create new economic options for the olive trade and reduce the environmental burden generated by these residual products. In recent decades, the comprehensive use of olive leaves has been the focus of increasing interest [7,8]. For example, olive leaf flour has been used as an ingredient for the development of healthy crackers [9], and olive leaf extracts have been marked as natural antioxidants to improve the stability of edible oils [10] and to extend the shelf-life of gluten-free bread [11]. In summary, olive leaves have a myriad of potential applications in value-added commercial products, such as in food ingredients, nutraceuticals, pharmaceuticals, and cosmetics.

Phenolic compounds represent one of the major classes of active compounds in plants; they are the main contributors to plant bioactivity [1,12]. The bioactivity of olive leaf extracts appears to be partly related to the antioxidant activity and various phenolic compounds in the leaves [13,14]. The composition, concentration, and antioxidant behavior of the phenolic compounds may affect the applications of olive leaves. Several factors can affect the phenolic profiles of olive leaves, such as cultivar/genotype, developmental stage, climate, season, and subsequent processing, such as the drying conditions, temperature, light, and oxygen exposure [15,16,17,18,19]. Among these, the cultivar/genotype will significantly affect the type and concentration of the phenolic compounds. In a previous study, the phenolic spectra of 15 olive leaf varieties were characterized by high-performance liquid chromatography (HPLC) coupled with electrospray ionization and quadrupole time-of-flight mass spectrometry, and the results showed that the types and concentrations of phenolic substances varied greatly among the varieties [15]. In another study, the qualitative and quantitative analyses of phenolic compounds in nine olive leaf genotypes showed significant differences in phenolic compound concentrations [20]. In 1956, olive trees were introduced into China from the Mediterranean [2], and, after 55 years of trial and cultivation, they have become a significant part of the agroindustry in China, with a total olive cultivation area of 80,000 hm^2^ [21]. Although several studies have investigated the phenolic concentration and antioxidant activities of olive leaves, the phytochemical compounds and antioxidant activities of China-grown olive leaves have not been systematically studied, and information on the contributions of individual phytochemicals of olive leaves on their antioxidant activity is limited.

To our knowledge, oil content is the most important economic factor for the characterization of olive cultivars [22,23]. Accordingly, it is indubitably important to take into account not only phytochemical profiles in leaves but also oil content in the fruits for the comprehensive utilization of olive leaves among different cultivars. In the present study, the phytochemical compositions and antioxidant activities of olive leaves from 32 cultivars grown in China, including nine high-oil-content (>20%), 12 medium-oil-content (16–20%) and 11 low-oil-content (<16%) cultivars, were systematically investigated. Moreover, olive cultivar evaluation from both oil contents and phytochemical profiles in leaves was conducted simultaneously for the first time. Furthermore, the relationships between the individual phytochemicals and antioxidant capacities of the leaves were analyzed to define which bioactive compounds were responsible for antioxidant activity. The results of this work may be useful for valorizing olive leaves as value-added functional ingredients for functional foods, cosmetics, and medicine.

## 2. Materials and Methods

### 2.1. Chemicals and Reagents

All chemicals and reagents used in this study were of analytical grade unless stated otherwise. Trolox, gallic acid, Folin–Ciocalteu phenol reagent, 2,2-diphenyl-1-picrylhydrazyl (DPPH), 2,2-azino-bis (3-ethylbenzothiazoline-6-sulfonic acid) diammonium salt (ABTS), and 2,4,6-tris (2-pyridyl)-s-triazine (TPTZ) were purchased from Sigma-Aldrich (St. Louis, MO, USA). Standard compounds such as hydroxytyrosol, esculin, taxifolin, luteolin, quercetin, kaempferol, apigenin, chlorogenic acid, plantamajoside, rutin, eriodictyol, tiliroside, apigenin-7-O-neohesperidoside, apigenin-7-O-glucoside, luteolin-7-O-glucoside, oleuropein, secoxyloganin, asiatic acid, oleanonic acid, maslinic acid, corosolic acid, oleanolic acid, and ursolic acid were purchased from Yuanye Bio-Technology Co., Ltd. (Shanghai, China). For chromatography analysis, HPLC-grade acetonitrile and acetic acid were purchased from Merck (Darmstadt, Germany) and Alfa Aesar (Shanghai, China), respectively. Ultrapure water supplied by a Milli-Q system (Millipore, Bedford, MA, USA) was used throughout the experiments.

### 2.2. Plant Material Preparation

Thirty-two olive cultivars were selected for investigation. All cultivars were planted in the research garden of the Institute of Crops and Nuclear Technology Utilization at the Zhejiang Academy of Agricultural Sciences, China under the same agronomic and environmental conditions. Details and pictures of the olive leaves are summarized in Table 1 and Figure S1. The samples comprised 20 olive oil cultivars, 3 table olive cultivars, and 7 olive cultivars which could be used for both olive oil extraction and table olive production. The samples also included two rootstock autochthonous cultivars: OL20 (*O. europaea* subsp. Cuspidata isolate Yunnan) and OL27 (Zhonglan). We classified all 32 olive cultivars into high-oil-content (>20%), medium-oil-content (16–20%) and low-oil-content (<16%) cultivars, according to the database (URL: www.oleadb.it, accessed on 25 January 2022) and our own data.

The olive leaf samples were manually picked between mid-November and mid-December 2020. The moisture contents of the olive leaf samples ranged from 43.27% for OL27 (Largueta cultivar) to 58.59% for OL31 (Morcona cultivar; Table 1), according to the Chinese National Standard GB5009.3-2016 (Determination of water in foods). The fresh olive leaves were oven-dried at 105 °C until a safe storage moisture content was reached. The dried leaves were then ground using a grinder, passed through a 60-mesh (250 μm) sieve to obtain a fine powder, and then stored in vacuum-sealed bags at −20 °C until extraction.

### 2.3. Sample Extraction

The dried olive leaf powder (1.00 g ± 0.01 g) from each cultivar was fully mixed with 10 mL of 70% ethanol solution and then extracted under continuous sonication (40 kHz) for 30 min at 50 °C. The leaf extracts were centrifuged at 5000 rpm for 10 min, and the supernatant was collected. The extraction processes were repeated three times. All supernatants were combined and diluted to 50 mL. Then, the total flavonoid content (TFC), total phenolic content (TPC), and antioxidant activities of the extracts were determined. Prior to phytochemical profile analyses, the extracts were filtered with a 0.22 μm syringe filter.

### 2.4. Phytochemical Profiling

#### 2.4.1. Identification of the Phytochemical Compounds via HPLC–Electrospray Ionization–Tandem Mass Spectrometry

The phytochemical compounds in the olive leaves were identified using an Agilent 1200LC system coupled with a Thermo Finnigan LCQ DECA mass spectrometer equipped with an electrospray source. Chromatographic separation was performed using a ZORBAX SB-C18 column (4.6 × 250 mm, 5 μm, Agilent Technologies, Savage, MD, USA) at a flow rate of 1.0 mL/min. The mobile phases were acetic acid (1%)/deionized water (mobile phase A) and acetic acid (1%)/acetonitrile (mobile phase B). The elution conditions were as follows: 0–11 min, 10–25% B; 11–16 min, 25–28.5% B; 16–40 min, 28.5–90% B; 40–50 min, 90% B; 50–55 min, 90–10% B; and 55–60 min, 10% B. The flow rate was 1 mL/min. Analyses were performed with scans from *m/z* 125 to 1200 Da in negative and positive ion modes. The peaks were identified using an Xcalibur Qual Browser by comparing the molecular ions, fragmentation, and relative retention times with the literature data and reference compounds.

#### 2.4.2. Quantification of Individual Phenolics by HPLC Coupled with Diode Array Detection

Individual phenolic compounds were quantitatively analyzed using a ZORBAX SB-C18 column (4.6 × 250 mm, 5 μm) in a Shimadzu LC-2030C HPLC system (Kyoto, Japan). The elution conditions were the same as those used for qualitative analysis, and the peaks were detected at 254, 280, and 320 nm. The peaks were then quantitatively analyzed by LabSolutions HPLC software using the calibration curves of the corresponding standards or a compound that contained a similar aglycone. The phenolics standards were dissolved in 80% methanol to obtain a concentration of approximately 1.0 mg/mL. The standard solution mixture was divided into five gradients using an approximately two-fold dilution process, and the five gradients were then used to prepare the calibration standards. Chromatographic separation was the same as the process used for the olive extracts.

#### 2.4.3. Quantification of Triterpenic Acids by HPLC Coupled with Diode Array Detection

The standards of asiatic acid, oleanonic acid, maslinic acid, corosolic acid, oleanolic acid, and ursolic acid were dissolved in 80% methanol to obtain a concentration of approximately 1.0 mg/mL. The standard solution mixture was then divided into five gradients using an approximately two-fold dilution process; the gradients were then used for the preparation of the calibration standards. The triterpene concentrations in the olive leaves were determined using a Shimadzu LC-2030C HPLC system coupled with a ZORBAX SB-C18 column. The mobile phases were acetic acid (1%)/deionized water (phase A) and acetic acid (1%)/acetonitrile (phase B), and the isocratic elution flow rates were 9% (A) and 91% (B). Then, the absorbance at 210 nm was detected. The spectral peaks were quantitatively analyzed with LabSolutions HPLC software using the calibration curves of the corresponding standards.

### 2.5. TFC

The TFC in the olive leaves was determined according to the aluminum chloride colorimetric method, using rutin as the standard [24]. First, 1 mL of the extracted samples was transferred into a 10 mL calibrated test tube, and 0.4 mL of 5% sodium nitrite solution was added. Then, the mixture was shaken for effective mixing and left to stand for 6 min. Afterward, 0.4 mL of 10% aluminum nitrate solution was added to the mixture. Then, the mixture was shaken and left to stand for 6 min. Finally, 4 mL of 5% sodium hydroxide solution and 10 mL of water were successively added to the mixture, and the mixture was shaken and left to stand for 15 min. Afterward, the absorbance value at 510 nm was measured. The TFC is expressed as mg rutin equivalent per gram of dry weight (mg RE/g dw).

### 2.6. TPC

The TPC was assessed according to the Folin–Ciocalteu method, using gallic acid as the standard [25]. First, 0.4 mL of each extract was transferred into a 25 mL graduated test tube, and 1 mL of the Folin–Ciocalteu reagent was added. After the mixture was shaken for effective mixing, the reagent was left to stand for 3–4 min. Then, 5 mL of 7.5% sodium carbonate solution was added, followed by distilled water, which was added to the different mixtures to achieve a constant volume of 25 mL. The mixtures were bathed in water at 40 °C for 30 min, and the absorbance at 765 nm was measured. The TPC is expressed as mg gallic acid equivalent per gram of dry weight (mg GAE/g dw).

### 2.7. Antioxidant Activity

The antioxidant activity of the olive leaf extracts was determined by the standard DPPH, ABTS, and ferric reducing/antioxidant power (FRAP) assays according to the methods established by Martinović and Cavoski [26] with some minor modifications. The results were calculated from the Trolox calibration curve and are expressed as mg Trolox equivalent per gram of dry weight (mg TE/g dw).

For the DPPH free radical scavenging assay, 0.1 mL of each extract was transferred into a test tube, and 3.9 mL of 0.1 mmol/L DPPH reaction solution was added. The reaction proceeded in the dark for 30 min. Then, the absorbance at 517 nm was measured.

For the ABTS assay, ABTS stock solutions including 7 mM ABTS and potassium persulfate (K_2_S_2_O_8_, 2.45 mM) were prepared. The ABTS stock solution was diluted with ethanol to obtain an absorbance of 0.70 ± 0.02 at 734 nm. Then, 3.9 mL of ABTS+ working solution and 0.1 mL of extracts were mixed for 6 min in the dark. Afterward, the absorbance was measured at 734 nm relative to the reagent blank (ethanol).

For the FRAP assay, 300 mM acetate buffer (pH 3.6), 10 mM TPTZ solution in 40 mmol/L HCl, and 20 mM ferric chloride were mixed at a ratio of 10:1:1 to prepare the FRAP reagent. Subsequently, 0.5 mL of extracts and 4.5 mL of FRAP reagent were transferred into a vial and incubated at 37 °C for 30 min. Then, the absorbance was measured at 593 nm.

### 2.8. Statistical Analysis

All experiments were conducted at least three times, and the results are expressed as mean ± standard deviation. Statistical analyses were performed using IBM SPSS Software 21 (Chicago, IL, USA). The parametric analysis of variance (ANOVA) was conducted by multifactor analysis of variance and post hoc Duncan’s multiple range test, where *p* < 0.05 was considered significant. Pearson’s correlation scatter plots between antioxidant activity (DPPH, ABTS, and FRAP), TPC, and TFC were constructed using GraphPad Software 7 (San Diego, CA, USA). The relationships between the concentrations of the 32 phenolics and 5 variables (TPC, TFC, DPPH, ABTS, and FRAP) were obtained using Spearman’s rank correlations, and a correlation heatmap was obtained using the vegan package in R software (version 3.1.2). Principal component analysis (PCA) was conducted on the mean values of the 32 phenolics and 5 variables (TPC, TFC, DPPH, ABTS, and FRAP) using SIMCA-P software (Umetrics, Umea, Sweden).

## 3. Results and Discussion

### 3.1. Identification and Quantification of Phytochemical Compounds

**Figure 1:**
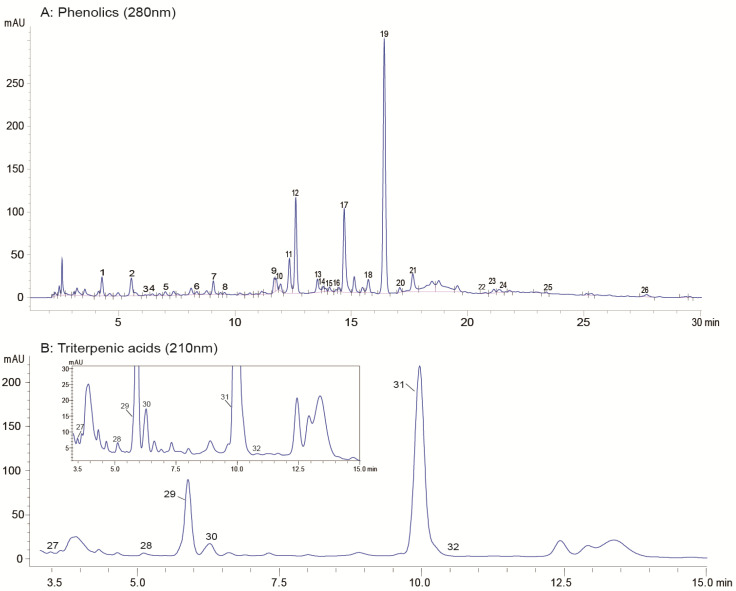
HPLC base peak chromatograms of the olive leaf extracts: (**A**) phenolics: (1) loganic acid; (2) hydroxytyrosol; (3) esculin; (4) taxifolin-3-glucoside; (5) chlorogenic acid; (6) secoxyloganin; (7) luteolin-3′,7-di-O-glucoside; (8) plantamajoside; (9) rutin; (10) luteolin rutinoside; (11) quercetin-3-O-glucoside; (12) luteolin-7-O-glucoside; (13) apigenin-7-O-neohesperidoside; (14) taxifolin; (15) quercetin 3-O-rhamnoside; (16) apigenin-7-O-glucoside; (17) kaempferol-7-O-glucoside; (18) luteolin-4′-O-glucoside; (19) oleuropein isomer 1; (20) oleuropein isomer 2; (21) oleuropein isomer 3; (22) eriodictyol; (23) luteolin; (24) quercetin; (25) kaempferol; (26) hispidulin; (**B**) triterpenic acids: (27) asiatic acid; (28) oleanonic acid; (29) maslinic acid; (30) corosolic acid; (31) oleanolic acid; and (32) ursolic acid.

In this study, 32 phytochemical compounds were identified (Table 2 and Figure 1), consisting of flavonoids (17), iridoids (5), hydroxycinnamic acids (2), triterpenic acids (6), simple phenols (1), and coumarins (1). The identified phytochemical compounds showed no differences among the 32 cultivars. Most of the reported data on olive leaves indicated that phenolics are the major biologically active compounds in olive leaves [1,12]. In recent decades, these compounds have attracted attention for human health purposes, due to their notable antioxidant activities through single-electron and hydrogen atom transfer [27]. The phenolic profiles of olive leaves previously detected in several common cultivars, such as Negrinha do Freixo, Hojiblanca, and Cornicabra, were slightly different from those identified in this work; however, the major class of phenolics (i.e., flavonoids, iridoids, simple phenols) exhibited the same profiles as those reported in the literature [28,29].

#### 3.1.1. Flavonoid Derivatives

Flavonoids represent one of the most diverse groups of phenolic compounds in olive leaves [28]. Plant-based flavonoids have strong antioxidant, antimicrobial, and anti-hyperglycemic potentials because of their ability to scavenge free radicals, kill many bacterial strains, and inhibit starch-digesting enzymes [33]. Our results showed that most of the flavonoids occurred in glycosylated forms in the olive leaves (e.g., luteolin-3′,7-di-O-glucoside, rutin, luteolin rutinoside, luteolin-7-O-glucoside, kaempferol-7-O-glucoside, quercetin-3-O-glucoside, taxifolin-3-glucoside, and apigenin-7-O-neohesperidoside). Moreover, six flavonoids were in the form of aglycones (i.e., luteolin, quercetin, taxifolin, eriodictyol, kaempferol, and hispidulin). The total quantities of glycosylated flavonoids (4.58–17.26 mg/g dw) were approximately 10 times those of the corresponding flavonoid aglycones (0.34–2.28 mg/g dw; Table S1).

**Figure 2:**
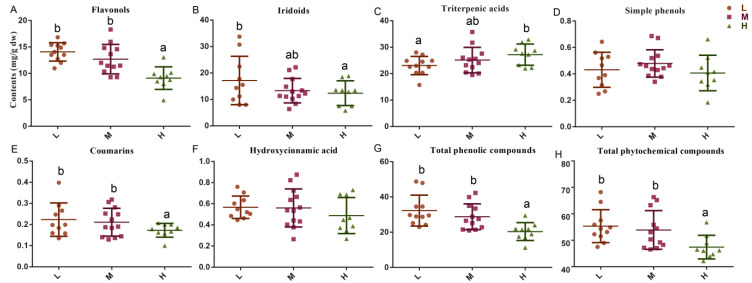

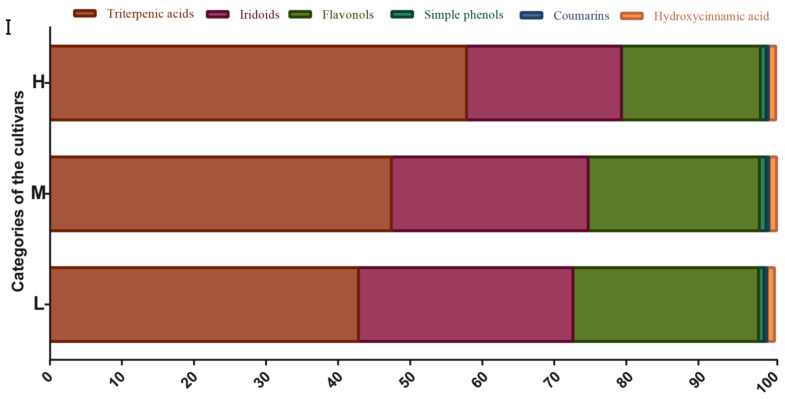
Compositions of the total quantity of (**A**) flavonols, (**B**) iridoids, (**C**) triterpenic acids, (**D**) simple phenols, (**E**) coumarins, (**F**) hydroxycinnamic acid, (**G**) total phenolic compounds, and (**H**) total phytochemical compounds in the olive leaf extracts from 32 cultivars and (**I**) distribution of the corresponding total content of the six phytochemicals in each category. For each category, the different letters indicate a significant difference (*p* < 0.05). H, M, and L represent high-oil-content (>20%) olive cultivars, medium-oil-content (16–20%) olive cultivars, and low-oil-content (<16%) olive cultivars, respectively.

According to previous studies, flavonoid expression in olives leaves was predominately driven by genetic and environmental factors [28], and the results of this study showed considerable differences among the cultivars. In all studied cultivars, luteolin-7-O-glucoside and kaempferol-7-O-glucoside were the most abundant flavonoids, with quantities of 1.20–7.00 and 1.49–5.32 mg/g dw, respectively; they were most abundant in cultivar OL10 (Ascolana tenera) and least abundant in OL1 (Bouteillan). Other abundant flavonoids included rutin, which was the most abundant in OL12 (Koroneiki, 1.48 mg/g dw), OL5 (Nocellara del belice, 1.45 mg/g dw), and quercetin-3-O-glucoside, which was most abundant in OL5 (1.14 mg/g dw) and OL7 (I-79, 1.12 mg/g dw). Further analysis was conducted from the oil content viewpoint, which has been one of the most important economic factors for the characterization of olive cultivars [22,23], in order to discriminate between the different cultivars. As shown in Figure 2A, the mean total quantities of flavonoids (TQFs) in the leaves of the olive cultivars with high, medium, and low oil contents were 9.07, 12.69, and 14.07 mg/g dw, respectively. Overall, six varieties with low or medium oil content showed relatively higher TQF values (>15 mg/g dw), namely, OL5 (M, 18.29 mg/g dw), OL9 (L, O. europaea subsp. Cuspidata isolate Yunnan, 16.81 mg/g dw), OL10 (M, 15.97 mg/g dw), OL2 (L, Fecciaro, 15.68 mg/g dw), OL7 (L, 15.39 mg/g dw), and OL11 (L, Zhonglan, 15.26 mg/g dw). However, the high-oil-content cultivar, OL1, exhibited the lowest TQF value at only 4.92 mg/g dw. In the present study, we assessed olive cultivars from both oil contents and useful by-products, simultaneously. Anastasiu et al. also proposed a selection criterion based on both oil productivity and the oil iodine value for linseed (*Linum usitatissimum* L.) which proved highly efficient to rank linseed cultivars from both a quantitative and qualitative viewpoint [34]. Consequently, cultivar evaluation from different viewpoints in a simultaneous manner may provide novel ideas for the efficient utilization of agricultural resources.

#### 3.1.2. Iridoid Derivatives

Five iridoid compounds were identified: loganic acid, secoxyloganin, and oleuropein and its isomers. Iridoids and their related subclass secoiridoids are rarely found in edible plants; however, they have been frequently reported as a major class of phenolics in olive fruits and leaves [3]. Furthermore, most of the observed antimicrobial, antiviral, antioxidant, antihypertensive, and antitumoral properties of olive leaf extracts have been attributed to secoiridoids, particularly oleuropein [1]. Oleuropein and its isomers were the predominant polyphenols in all studied cultivars (Table S2), especially in the two autochthonous cultivars (OL3 [Frantoio selection] and OL9: 32.05 and 29.27 mg/g dw, respectively), whose oleuropein concentrations were approximately six times those of the cultivars with the lowest oleuropein concentrations (OL1 and OL26 [Olivon de Roda]: 5.21 and 5.25 mg/g dw, respectively). Moreover, loganic acid and secoxyloganin were also abundant in all cultivars, and they were most abundant in OL24 (Castellana, 1.23 mg/g dw) and OL5 (1.69 mg/g dw), respectively. Similarly, iridoid compound content varied greatly among the three categories in terms of oil content (Figure 2B, *p* < 0.05). The cultivars with high oil content had the lowest total iridoid content of 10.22 mg/g dw, while the cultivars with low oil content had the highest value of 16.88 mg/g dw. Cecchi et al. also found that the oil content and oleuropein quantity during ripening were cultivar-dependent, but they did not highlight strong correlations between the oil content and phenolic compounds [35].

#### 3.1.3. Terpene Derivatives

In addition to flavanols and iridoids, triterpenic acids (i.e., asiatic, oleanonic, maslinic, corosolic, oleanolic, and ursolic acid) have also been found to be a major class of bioactive compounds in olive leaves. Table olives and olive oil have been reported to be rich in maslinic and oleanolic acids, and small quantities of ursolic acid have been detected in olive oils [16,36]. In olive products, the maslinic acid concentration ranges from 287.1 ± 66.6 in the Manzanilla variety, 1318.4 ± 401.0 mg/kg in Arbequina table olives, and from 64.2 ± 8.1 to 193.9 ± 14.0 mg/kg in olive oils [37]. Despite the presence of triterpenic acids in olive fruits, data on the quantities of these substances in olive leaves are scarce. In this study, we showed the presence of important levels of triterpenic acids in olive leaves. The overall concentration of triterpenic acids ranged from 15.72 mg/g dw (OL9; Table S2) to 35.75 mg/g dw (OL16, Picholine), and maslinic and oleanolic acids were more abundant than asiatic, oleanonic, corosolic, and ursolic acids in all of the studied varieties. Unlike flavonoids and iridoids, the leaves of olive cultivars with high oil content contained significantly higher mean triterpenic acid content (27.19 mg/g dw) than the other two categories (25.14 and 23.08 mg/g dw for medium- and low-oil-content cultivars, respectively; Figure 2C). In addition, triterpenic acids were the dominant phytochemicals (accounting for 57.65%) in the olive leaves of the high-oil-content categories (Figure 2I). These results suggested that cultivars with high oil content could be a good choice for the extraction of triterpenic acids, which could serve as potential nutraceuticals for valorizing olive leaves. Recently, triterpenic acids, especially oleanolic and maslinic acids, were demonstrated to possess multiple health-protective activities, including anti-diabetic, anti-inflammatory, cardioprotective, and anti-tumoral properties [36,38].

#### 3.1.4. Simple Phenol, Coumarins, and Hydroxycinnamic Acids

We identified one simple phenol (hydroxytyrosol), one coumarin (esculin), and two hydroxycinnamic acids (plantamajoside and chlorogenic acid) in the olive leaf extracts of all the studied varieties. Previous research has also identified coumarins and hydroxycinnamic acids in olive leaves [39]. Not only was hydroxytyrosol the main simple phenol in olive leaves, but it was also vital for secoiridoid formation [1]; its concentration ranged from 0.18 to 0.69 mg/g dw. Additionally, the hydroxytyrosol, esculin, plantamajoside, and chlorogenic acid contents in the different cultivars varied greatly (Table S2); however, the differences between the three categories were not significant (Figure 2D–F).

### 3.2. TPC, TFC, and Antioxidant Activities

TPC and TFC evaluation is a rapid, sensitive, and robust method for assessing the quantitative composition of biologically active compounds. The TFC and TPC of the olive leaves were determined through the aluminum chloride colorimetric method and Folin–Ciocalteu assay. No significant differences in the TPC and TFC were found among the three categories in terms of oil content (Figure S2A,B), even though substantial changes were found among the different cultivars. As shown in Table 3, the leaves of the 32 olive cultivars exhibited slight differences in TPC, ranging from 15.13 ± 0.19 to 17.49 ± 0.12 mg GAE/g dw. OL31 (Morcona) exhibited the highest TPC value in the leaf extracts, followed by OL32 (Gentile di chieti), OL4 (Manzanilla), and OL20 (Canino), whereas OL23 (Nevadillo fino) exhibited the lowest TPC value. Gullon et al. reported a TPC of 12.36–27.54 mg/g in dried olive mill leaves [40]. Thus, the TPC values obtained in this study were within the literature-reported range.

The TFCs significantly differed between the cultivars. Among the studied cultivars, OL9 exhibited the highest TFC value in the leaf extracts (176.30 ± 20.04 mg RE/g dw), followed by OL3 (174.22 ± 16.01 mg RE/g dw), whereas OL15 (Nikitskii I) and OL1 exhibited the lowest amount of TFCs (71.89 ± 16.82 and 72.79 ± 24.00 mg RE/g dw, respectively). The TFCs obtained in this study were substantially greater than those reported by Gullon et al. (11.52 to 52.82 mg RE/gdw) [41] and comparable to those obtained for cultivar Picual leaf extracts (approximately 66.7 mg RE/g dw) [12]. Multiple factors, including extraction treatment, harvesting season, climate, variety, and ripening degree, can affect the composition and concentrations of bioactive chemicals [19] in leaf extracts. Thus, it is difficult to compare the data obtained in this study with data in the literature.

The antioxidant activities of the olive leaf extracts from the 32 cultivars were evaluated by three complementary methods: DPPH, ABTS, and FRAP assays, which were based on radical scavenging ability and reducing power. As shown in Table 3, no significant differences (*p* > 0.05) in DPPH-based antioxidant activity were determined between the cultivars. Antioxidant activities varied between 171.10 ± 6.95 and 180.12 ± 3.50 mg TE/g dw. However, the olive leaves of the investigated varieties showed statistically significant ABTS- and FRAP-based antioxidant activities, and their overall trends were similar to those for the total quantities of individual phenolics and TFC. Similarly, OL3 and OL14 were in the low-oil-content category and exhibited the highest ABTS values (758.01 ± 16.54 and 710.64 ± 14.58 mg TE/g dw, respectively), while the high-oil-content cultivar, OL1, exhibited the lowest ABTS value (279.71 ± 17.57 mg TE/g dw). For the FRAP values, OL9 (L), OL5 (M), and OL3 (L) exhibited the highest values, at 1228.29 ± 23.95, 1139.86 ± 17.05, and 1099.99 ± 14.30 mg TE/g dw, respectively, which coincided with the maximum TFC value. OL1 also had the lowest FRAP value of 385.13 ± 50.20 mg TE/g dw. As expected, the high-oil-content cultivars had the lowest antioxidant values (Figure S2D,E). In addition, the variations between the results of the DPPH, ABTS, and FRAP assays were attributed to the use of different radicals in the different methods, as the various compounds reacted differently with the employed radicals [42]. Consequently, our findings showed that combining antioxidant capacity assays was more accurate than using only the DPPH assay.

### 3.3. Multivariate Data Analysis

#### 3.3.1. Correlation Analysis

**Figure 3:**
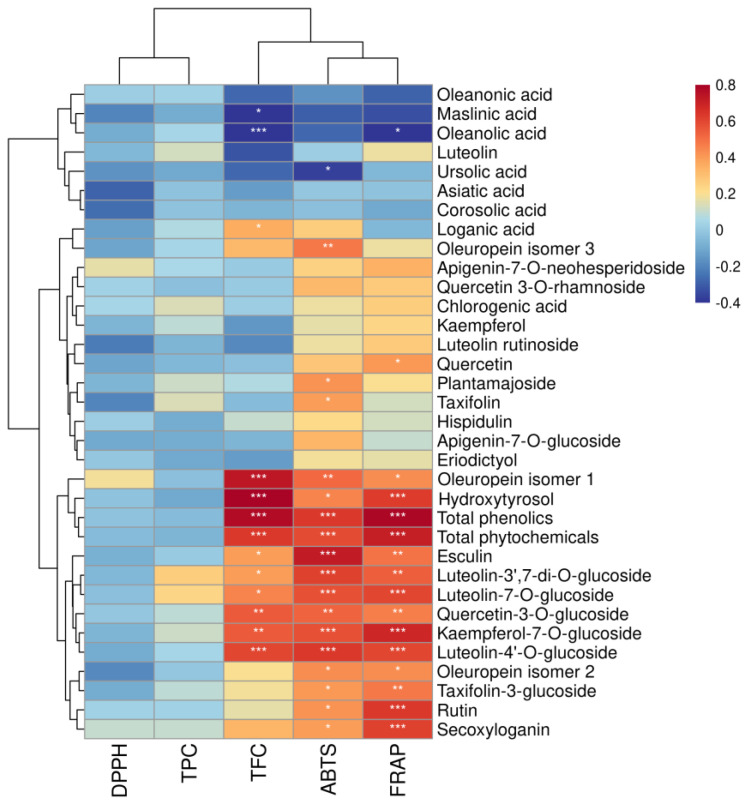
Heatmap of Spearman’s rank correlations between the individual phytochemicals and five variables (TPC, TFC, DPPH, ABTS, and FRAP) of the olive leaf extracts. Significance levels are indicated as follows: *: *p* < 0.05; **: *p* < 0.01; ***: *p* < 0.001.

The correlations between antioxidant activity (DPPH, ABTS, and FRAP assays) and TPC, TFC, and the concentrations of the individual phytochemicals of olive leaves were determined using Pearson’s and Spearman’s rank correlation analyses. No significant correlations were found between TPC and antioxidant activity (DPPH, ABTS, and FRAP assays; Figure S3); however, the total quantities of phenolic compounds were highly correlated with the ABTS-based (r = 0.6389; *p* < 0.001) and FRAP-based antioxidant activities (r = 0.7808; *p* < 0.001; Figure 3). This discrepancy was attributed to the non-specificity of the colorimetric method for TPC [43]. Furthermore, the TFCs also showed significant (*p* < 0.001; Figure S3) positive correlations with the ABTS-based (r = 0.6856) and FRAP-based antioxidant activities (r = 0.6236). Flavonoids, the major groups of polyphenols in olive leaves, have shown to be major contributors to the demonstrated antioxidant activity of olive leaf extracts, due to their free hydroxyl groups and catechol structures [44]. The correlation between antioxidant activity and the concentrations of individual phenolics confirmed that the synergistic effect of a larger number of flavonoids led to the antioxidant behavior of the olive leaves. Kaempferol-7-O-glucoside, luteolin-7-O-glucoside, luteolin-3′,7-di-O-glucoside, luteolin-4′-O-glucoside, and rutin showed strong significant correlations (r = 0.5244–0.7627; *p* < 0.001) with the ABTS and FRAP values. In addition to the flavonoids, oleuropein, secoxyloganin, esculin, and hydroxytyrosol concentrations also showed significant (*p* < 0.01) positive correlations with antioxidant activities for the ABTS and FRAP values. Despite the high concentrations of oleuropein identified in the olive leaf extracts, oleuropein showed weaker correlations with antioxidant activity than hydroxytyrosol. A previous study reported that the antioxidant activity of oleuropein was mainly due to the aglycones (i.e., hydroxytyrosol moietyin) in its structure [13]. The antioxidant activity of plant-derived samples has shown to be a complicated action that could be synergistically promoted by the various phenolic compounds in the plant [42]. Similarly, the antioxidant behavior of olive leaf extracts may largely depend on the interactions of bioactive chemicals, including flavonoids, secoiridoids, and hydroxytyrosol [13,44]. Nevertheless, modern analytical techniques such as DPPH•/ABTS•+/FRAP, coupled with ultra-high-performance liquid chromatography-high-resolution mass spectrometry, should be further used to identify and screen the main antioxidant compounds in olive leaves [45].

#### 3.3.2. PCA

**Figure 4:**
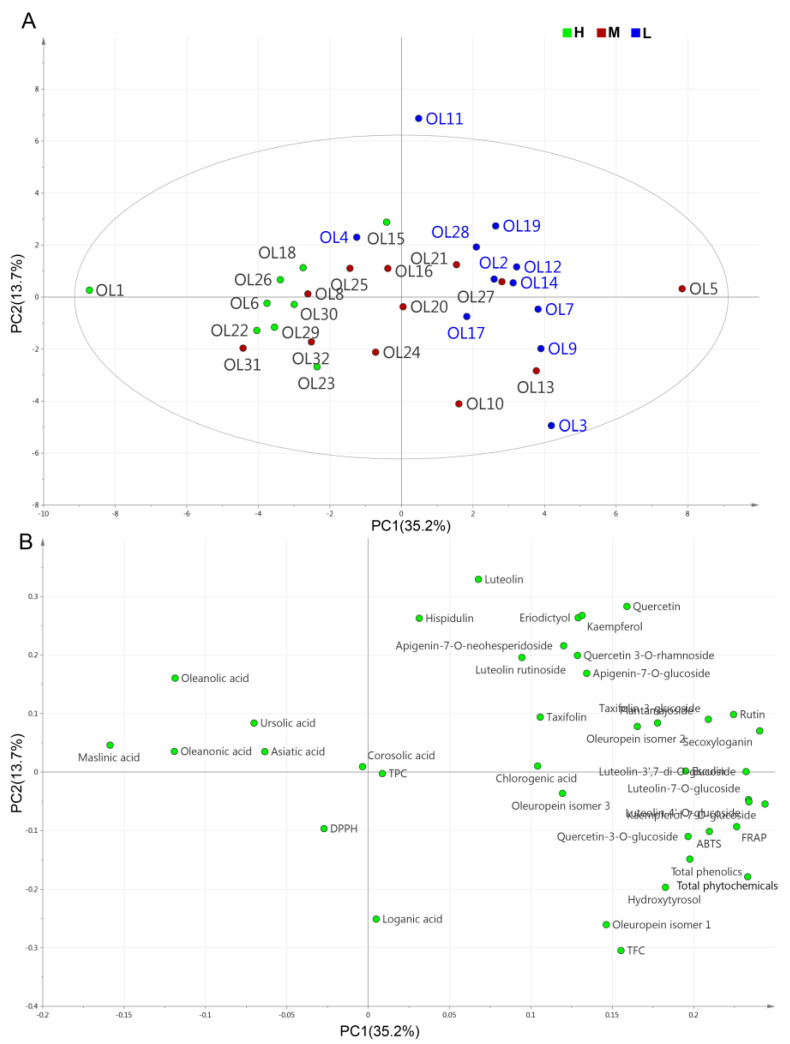
PCA results based on the mean values of 32 phytochemicals and five variables (TPC, TFC, DPPH, ABTS, and FRAP): (**A**) score plot and (**B**) loading plot. H, M, and L represent high-oil-content (>20%) olive cultivars, medium-oil-content (16–20%) olive cultivars, and low-oil-content (<16%) olive cultivars, respectively.

Principal component analysis (PCA) has been widely used as an exploratory analysis to decrease the dimensionality of large datasets and to reflect similarities or differences among plant samples [24,43]. In the present study, the analysis was conducted using a complete dataset obtained from 32 cultivars (i.e., TPC, TFC, the concentrations of 32 phytochemical compounds, and the antioxidant activities of DPPH, ABTS, and FRAP). The first four principal components (PCs) with eigenvalues > 2 explained 64.8% of total variance (PC1 35.2%, PC2 13.7%, PC2 8.4%, and PC4 7.5%). As shown in Figure S4, the eigenvalue dropped significantly after the first two PCs. Thus, PC1 and PC2 were used to visualize the correlation between variables. The distribution of the 32 olive cultivars on a plane is shown in Figure 4A, and the contribution of each parameter is shown in the PCA loading plot (Figure 4B). As shown in the score plots, cultivars with medium oil content could not be differentiated as groups, due to their high variability, and were randomly distributed in Figure 4A. However, the first principal component separated the high-oil-content and low-oil-content cultivars. The variables that contributed most to PC1 (positive) were glycosylated flavonoids (e.g., rutin, luteolin-7-O-glucoside, luteolin-4′-O-glucoside, kaempferol-7-O-glucoside, and luteolin-3′,7-di-O-glucoside), oleuropein isomer 1, secoxyloganin, hydroxytyrosol, and antioxidant capacity parameters (ABTS and FRAP). In addition, triterpenic acids (i.e., oleanonic, maslinic, and oleanolic acids) had a strong negative influence on PC1. The leaves of the high-oil-content olive cultivars were positioned on the left side of the diagram and exhibited the lowest polyphenol concentrations and related antioxidant activities; however, they also had the highest triterpenic acid concentrations. Most of the low-oil-content cultivar leaves were concentrated on the right side (except for OL4) and exhibited the highest concentrations of glycosylated flavonoids, oleuropein, and hydroxytyrosol. These results were similar to the ANOVA results (Figure 2), which showed that the flavonoids, iridoids, and triterpenic acids were substantially different among the three categories in terms of oil content. In addition, analysis of the interdependencies between antioxidant activity (ABTS and FRAP) and individual polyphenols revealed that rutin, luteolin-7-O-glucoside, luteolin-4′-O-glucoside, kaempferol-7-O-glucoside, oleuropein, and hydroxytyrosol synergistically contributed to antioxidant activity, which was consistent with the correlation analysis results. In summary, the olive leaves from the varieties rich in these compounds could potentially provide high antioxidant activity and nutraceutical benefits.

## 4. Conclusions

This study evaluated the phytochemical profiles and antioxidant potential of olive leaves from 32 cultivars grown in China and revealed that olive leaves were excellent sources of flavonoids, iridoids, and triterpenic acids. TPC, TFC, and the individual phytochemical concentrations in the olive leaves varied considerably among the different cultivars, resulting in the various antioxidant activities of the different cultivars. Additionally, for the first time, we assessed olive cultivars for both oil contents and useful by-products from the leaves, simultaneously. Among the investigated cultivars, we found that leaves from the low-oil-content cultivars were characterized by high concentrations of glycosylated flavonoids, oleuropein, and hydroxytyrosol, whereas the high-oil-content olive cultivars were rich in triterpenic acids (i.e., oleanonic, maslinic, and oleanolic acid). Accordingly, it was suggested that olive leaves obtained from low-oil-content cultivars may be good candidates for flavonoid and oleuropein separation, whereas cultivars with high oil content may be suitable for the extraction of triterpenic acids. In future studies, it would be interesting to clarify the reason for the correlation between oil content and phytochemical compounds in leaves among different cultivars.

## Figures and Tables

**Table 1:** Summary of the 32 olive cultivars used in this study.

Code	Cultivar	Origin	Moisture Content%	Attitude	Oil Content
OL1	Bouteillan	France	45.05 ± 1.76 ^ab^	O/T	H
OL2	Fecciaro	Italy	54.83 ± 2.25 ^kl^	O	L
OL3	Frantoio selection	China	47.71 ± 1.20 ^de^	O	L
OL4	Manzanilla	Italy	47.16 ± 1.36 ^cde^	T	L
OL5	Nocellara del belice	Italy	54.77 ± 1.38 ^kl^	O/T	M
OL6	Picudo de Labata	Spain	44.66 ± 1.29 ^ab^	O	H
OL7	I-79	Italy	50.45 ± 1.56 ^fg^	O	L
OL8	Pendolino	Italy	56.56 ± 0.31 ^lm^	O	M
OL9	O. europaea subsp. Cuspidata isolate Yunnan	China	48.84 ± 0.83 ^ef^	R	L
OL10	Ascolana tenera	Italy	57.81 ± 2.26 ^n^	T	M
OL11	Zhonglan	China	48.23 ± 1.88 ^ef^	R	L
OL12	Koroneiki	Greece	52.12 ± 1.12 ^ij^	O	L
OL13	Arbequina	Spain	52.33 ± 1.30 ^ij^	O	M
OL14	Huaou 5	China	52.04 ± 0.50 ^ij^	O	L
OL15	Nikitskii I	Azerbaijan	52.16 ± 1.79 ^ij^	O/T	H
OL16	Picholine	France	51.08 ± 0.97 ^gh^	O/T	M
OL17	Chemlal de Kabylie	Algeria	49.02 ± 0.60 ^efg^	O	L
OL18	Hojiblanca	Spain	46.76 ± 2.50 ^bc^	O/T	H
OL19	Manzanilla sevillana	Spain	43.76 ± 0.86 ^a^	T	L
OL20	Canino	Italy	53.75 ± 1.35 ^jk^	O	M
OL21	Cipressino	Italy	55.12 ± 0.80 ^kl^	O	M
OL22	Rosciola	Italy	57.23 ± 1.02 ^mn^	O	H
OL23	Nevadillo fino	Spain	51.31 ± 0.31 ^hij^	O	H
OL24	Castellana	Spain	51.30 ± 1.20 ^hij^	O	M
OL25	Neral	Spain	53.72 ± 0.77 ^jk^	O	M
OL26	Olivon de Roda	Spain	47.89 ± 0.63 ^e^	O	H
OL27	Largueta	Spain	43.27 ± 1.74 ^a^	O	M
OL28	Manzanilla Greece	Spain	45.53 ± 0.59 ^ab^	O/T	L
OL29	Blanqueta	Spain	54.80 ± 0.82 ^kl^	O	H
OL30	Benizar	Spain	51.66 ± 1.35 ^ij^	O/T	H
OL31	Morcona	Italy	58.59 ± 0.42 ^n^	O	M
OL32	Gentile di chieti	Italy	58.23 ± 0.61 ^n^	O	M

O: olive oil cultivars; T: table olive cultivars; O/T: olive cultivars that can be used in both olive oil extraction and table olive production; R: rootstock cultivars; H, M, and L represent high-oil-content (>20%) olive cultivars, medium-oil-content (16–20%) olive cultivars, and low-oil-content (<16%) olive cultivars, respectively; ^a–n^ Means in the same column with unlike superscripts differ significantly (*p* < 0.05).

**Table 2:** Characterization of phenolic compounds from olive leaves via HPLC–ESI–MS.

No.	Proposed Compounds	Rt min	Molecular Formula	Ionization (ESI+/ESI−)	*m/z* Experimental	Class	Reference
1	Loganic acid	4.28	C_16_H_24_O_10_	[M-H]−	375.1	Iridoids	Alañón et al., 2020 [29]
2	Hydroxytyrosol	5.543	C_8_H_10_O_3_	[M-H]−	153.1	Simple phenols	Standard
3	Esculin	6.14	C_15_H_16_O_9_	[M-H]−	339.2	Coumarins	Standard
4	Taxifolin-3-glucoside	6.451	C_21_H_22_O_12_	[M-H]−	465.2	Flavonoids	Abaza et al., 2017 [30]
5	Chlorogenic acid	7.003	C_16_H_18_O_9_	[M-H]−	353.2	Hydroxycinnamic acid	Standard
6	Secoxyloganin	8.359	C_17_H_24_O_11_	[M-H]−	403.2	Iridoids	Standard
7	Luteolin-3′,7-di-O-glucoside	9.069	C_27_H_30_O_16_	[M-H]−	609.1	Flavonoids	Alañón et al., 2020 [29]
8	Plantamajoside	9.55	C_29_H_36_O_16_	[M-H]−	639.3	Hydroxycinnamic acid	Standard
9	Rutin	11.62	C_27_H_30_O_16_	[M-H]−	609.3	Flavonoids	Standard
10	Luteolin rutinoside	11.767	C_27_H_30_O_15_	[M-H]−	593.3	Flavonoids	Alañón et al., 2020 [29]
11	Quercetin-3-O-glucoside	12.342	C_21_H_20_O_12_	[M-H]−	463.2	Flavonoids	Standard
12	Luteolin-7-O-glucoside	12.613	C_21_H_20_O_11_	[M-H]−	447.4	Flavonoids	Standard
13	Apigenin-7-O-neohesperidoside	13.546	C_27_H_30_O_14_	[M-H]−	577.3	Flavonoids	Standard
14	Taxifolin	13.793	C_15_H_12_O_7_	[M-H]−	303.3	Flavonoids	Standard
15	Quercetin 3-O-rhamnoside	14.347	C_21_H_20_O_11_	[M-H]−	447.2	Flavonoids	Vinha et al., 2005 [31]
16	Apigenin-7-O-glucoside	14.47	C_21_H_20_O_10_	[M-H]−	431.4	Flavonoids	Alañón et al., 2020 [30]
17	Kaempferol-7-O-glucoside	14.697	C_21_H_20_O_11_	[M-H]−	447.2	Flavonoids	Standard
18	Luteolin-4’-O-glucoside	15.733	C_21_H_20_O_11_	[M-H]−	447.3	Flavonoids	Abaza et al., 2017 [30]
19	Oleuropein isomer 1	16.419	C_25_H_32_O_13_	[M-H]−	539.2	Iridoids	Standard
20	Oleuropein isomer 2	17.089	C_25_H_32_O_13_	[M-H]−	539.2	Iridoids	Abaza et al., 2017 [30]
21	Oleuropein isomer 3	17.647	C_25_H_32_O_13_	[M-H]−	539.2	Iridoids	Abaza et al., 2017 [30]
22	Eriodictyol	20.765	C_15_H_12_O_6_	[M-H]−	287.1	Flavonoids	Standard
23	Luteolin	21.133	C_15_H_10_O_6_	[M-H]−	285.5	Flavonoids	Standard
24	Quercetin	21.35	C_15_H_10_O_7_	[M-H]−	301.3	Flavonoids	Standard
25	Kaempferol	24.85	C_15_H_10_O_6_	[M-H]−	285.4	Flavonoids	Standard
26	Hispidulin	27.701	C_16_H_12_O_6_	[M-H]−	299.3	Flavonoids	Blasi et al., 2018 [32]
27	Asiatic acid	38.11	C_30_H_48_O_5_	[M-H]−	487.3	Triterpenic acids	Standard
28	Oleanonic acid	38.71	C_30_H_46_O_3_	[M+H]+	455.3	Triterpenic acids	Standard
29	Maslinic acid	39.64	C_30_H_48_O_4_	[M+H]+	473.5	Triterpenic acids	Standard
30	Corosolic acid	40.19	C_30_H_48_O_4_	[M+H]+	473.2	Triterpenic acids	Standard
31	Oleanolic acid	44.61	C_30_H_48_O_3_	[M-H]−	457.5	Triterpenic acids	Standard
32	Ursolic acid	44.33	C_30_H_48_O_3_	[M+H]+	457.3	Triterpenic acids	Standard

Rt: retention time.

**Table 3:** TPC, TFC, and antioxidant activities of olive leaf extracts from 32 cultivars.

Code	Oil Content	TPC (mg GAE/g dw)	TFC (mg RE/g dw)	DPPH (mg TE/g dw)	FRAP (mg TE/g dw)	ABTS (mg TE/g dw)
OL1	H	16.23 ± 0.23 ^b–h^	72.79 ± 24.00 ^ab^	176.99 ± 8.43	385.13 ± 50.20 ^a^	279.71 ± 17.57 ^a^
OL2	L	16.42 ± 0.15 ^c–i^	94.61 ± 10.87 ^a–c^	179.27 ± 4.47	765.99 ± 25.98 ^e–i^	541.13 ± 10.79 ^b–g^
OL3	L	16.87 ± 0.41 ^g–j^	174.22 ± 16.01 ^e^	178.62 ± 3.40	1099.99 ± 14.30 ^kl^	758.01 ± 16.54 ^l^
OL4	L	16.95 ± 0.40 ^h–j^	83.71 ± 15.19 ^ab^	178.56 ± 3.47	674.92 ± 62.07 ^b–g^	494.09 ± 32.17 ^bc^
OL5	M	16.57 ± 0.19 ^d–i^	127.97 ± 20.21 ^a–e^	176.54 ± 3.27	1139.86 ± 17.05 ^kl^	637.53 ± 8.43 ^g–k^
OL6	H	16.10 ± 0.19 ^b–g^	77.19 ± 20.20 ^ab^	180.12 ± 3.50	666.07 ± 12.74 ^b–g^	446.16 ± 16.42 ^b^
OL7	L	16.36 ± 0.17 ^b–i^	133.72 ± 12.19 ^b–e^	176.25 ± 2.20	952.77 ± 94.68 ^i–k^	612.91 ± 18.27 ^d–k^
OL8	M	15.60 ± 0.46 ^ab^	109.86 ± 9.37 ^a–e^	175.63 ± 1.20	836.55 ± 20.28 ^g–j^	519.86 ± 7.34 ^b–e^
OL9	L	15.91 ± 0.16 ^a–f^	176.30 ± 20.04 ^e^	180.22 ± 1.84	1228.29 ± 23.95 ^l^	623.75 ± 10.08 ^f–k^
OL10	M	16.71 ± 0.24 ^f–j^	132.60 ± 2.52 ^b–e^	174.14 ± 0.93	900.53 ± 83.56 ^h–j^	603.78 ± 27.91 ^d–j^
OL11	L	16.41 ± 0.25 ^b–i^	62.44 ± 26.05 ^a^	175.27 ± 0.90	738.49 ± 28.74 ^d–h^	526.05 ± 33.80 ^b–f^
OL12	L	16.34 ± 0.17 ^b–i^	111.50 ± 22.45 ^a–e^	173.98 ± 1.58	989.40 ± 21.17 ^jk^	598.43 ± 13.70 ^d–j^
OL13	M	16.23 ± 0.35 ^b–h^	156.56 ± 0.76 ^c–e^	177.23 ± 0.36	984.01 ± 17.04 ^jk^	670.33 ± 18.34 ^i–l^
OL14	L	15.81 ± 0.24 ^a–d^	116.71 ± 19.47 ^a–e^	176.26 ± 1.07	729.84 ± 16.91 ^d–h^	710.64 ± 14.58 ^kl^
OL15	H	16.69 ± 0.17 ^f–j^	71.89 ± 16.82 ^ab^	176.76 ± 0.95	666.17 ± 8.56 ^b–g^	609.78 ± 5.55 ^d–k^
OL16	M	16.09 ± 0.37 ^b–g^	98.13 ± 17.84 ^a–c^	174.91 ± 2.58	809.46 ± 27.34 ^f–j^	643.48 ± 19.09 ^g–k^
OL17	L	16.63 ± 0.27 ^e–i^	117.15 ± 25.21 ^a–e^	176.32 ± 0.72	755.47 ± 47.44 ^e–h^	603.15 ± 23.80 ^d–j^
OL18	H	16.85 ± 0.19 ^g–j^	100.20 ± 15.16 ^a–c^	177.01 ± 0.64	667.83 ± 20.57 ^b–g^	543.22 ± 9.74 ^b–g^
OL19	L	15.60 ± 0.05 ^ab^	109.34 ± 7.73 ^a–d^	171.10 ± 6.95	902.11 ± 46.12 ^h–j^	684.90 ± 3.00 ^j–l^
OL20	M	16.93 ± 0.29 ^h–j^	116.32 ± 9.32 ^a–e^	173.21 ± 1.50	709.83 ± 12.35 ^c–g^	624.14 ± 20.01 ^f–k^
OL21	M	16.26 ± 0.25 ^b–i^	97.38 ± 12.82 ^a–c^	175.51 ± 0.72	675.07 ± 28.56 ^b–g^	627.88 ± 16.66 ^f–k^
OL22	H	15.70 ± 0.18 ^a–c^	103.38 ± 1.98 ^a–c^	173.26 ± 2.44	710.58 ± 11.15 ^c–g^	584.71 ± 14.90 ^c–j^
OL23	H	15.13 ± 0.19 ^a^	125.42 ± 12.40 ^a–e^	175.32 ± 2.65	743.95 ± 13.34 ^d–h^	622.64 ± 13.13 ^e–k^
OL24	M	15.77 ± 0.19 ^a–d^	132.18 ± 18.98 ^b–e^	175.90 ± 2.87	637.54 ± 16.82 ^b–f^	594.99 ± 11.18 ^c–j^
OL25	M	16.70 ± 0.09 ^f–j^	77.72 ± 18.84 ^ab^	174.31 ± 0.70	516.82 ± 14.34 ^ab^	575.63 ± 18.34 ^c–i^
OL26	H	16.26 ± 0.16 ^b–i^	93.57 ± 10.43 ^a–c^	176.70 ± 0.81	516.90 ± 16.22 ^ab^	511.46 ± 13.69 ^b–d^
OL27	M	15.77 ± 0.27 ^a–d^	117.31 ± 19.59 ^a–e^	176.21 ± 0.41	620.80 ± 14.89 ^b–e^	631.50 ± 15.25 ^g–k^
OL28	L	16.11 ± 0.19 ^b–g^	108.21 ± 19.78 ^a–d^	176.74 ± 1.24	671.57 ± 36.85 ^b–g^	658.19 ± 19.80 ^h–l^
OL29	H	15.71 ± 0.38 ^a–c^	114.58 ± 8.76 ^a–e^	177.11 ± 0.46	505.07 ± 20.15 ^ab^	557.46 ± 23.16 ^c–h^
OL30	H	15.85 ± 0.08 ^a–e^	108.89 ± 3.29 ^a–d^	174.51 ± 0.66	557.54 ± 2.84 ^a–d^	563.59 ± 14.64 ^c–h^
OL31	M	17.49 ± 0.12 ^j^	102.23 ± 7.94 ^a–c^	177.22 ± 1.22	526.39 ± 17.86 ^a–c^	550.35 ± 7.54 ^c–g^
OL32	M	17.04 ± 0.24 ^ij^	115.23 ± 21.14 ^a–e^	176.06 ± 0.58	599.90 ± 9.56 ^b–e^	624.58 ± 23.24 ^f–k^

Note: Results are expressed as mean ± SD (n = 3). ^a–l^ Means in the same column with unlike superscripts differ significantly (*p* < 0.05). H, M, and L represent high-oil-content (>20%) olive cultivars, medium-oil-content (16–20%) olive cultivars, and low-oil-content (<16%) olive cultivars, respectively.

## Data Availability

All the data generated by this research are included in the article.

## Supplementary Materials

Figure S1: Leaves from the 32 investigated olive cultivars; Figure S2: TPC, TFC, and antioxidant activities of olive leaf extracts from 32 cultivars; Figure S3: Pearson’s correlation scatter plot of the relationships between (A) DPPH and TPC, (B) FRAP and TPC, (C) ABTS and TPC, (D) DPPH and TFC, (E) FRAP and TFC, and (F) ABTS and TFC; Figure S4: Eigenvalues for principal component analysis (PCA); Table S1: Quantitative amounts of flavonols (mg/g dw) in olive leaf extractsfrom 32 different cultivars; Table S2: Quantitative amounts of simple phenols, coumarins, iridoids, triterpenic acids, and hydroxycinnamic acid (mg/g dw) in olive leaf extracts from 32 different cultivars.
