# Supplementary material for: Comparative Evaluation of the Phytochemical Profiles and Antioxidant Potentials of Olive Leaves from 32 Cultivars Grown in China
Source: Molecules. 2022 Feb 15;27(4):1292. doi: 10.3390/molecules27041292 (PMC8878581; doi:10.3390/molecules27041292)
Supplement: Supplementary file 1 [file molecules-27-01292-s001.zip › Supplementary materials.pdf]

Comparative evaluation of the phytochemical profiles andantioxidant potentials of olive leaves from 32 cultivarsgrown in China

Chengcheng Zhang[1], Xiaoting Xin[1], Jianming Zhang[1], Shenlong Zhu[2], Erli Niu[2], Zhongjing Zhou[3] and Daqun Liu[1,*]

[1] Food Science Institute, Zhejiang Academy of Agricultural Sciences, Hangzhou 310021, China

[2] Institute of Crop and Nuclear Technology Utilization, Zhejiang Academy of Agricultural Sciences, Hangzhou 310021, China

[3] State Key Laboratory for Managing Biotic and Chemical Threats to the Quality and Safety of Agro-products, Zhejiang Academy of Agricultural Sciences, Hangzhou 310021, China

* Corresponding author:Daqun Liu, E-mail: liudaqun@zaas.ac.cn

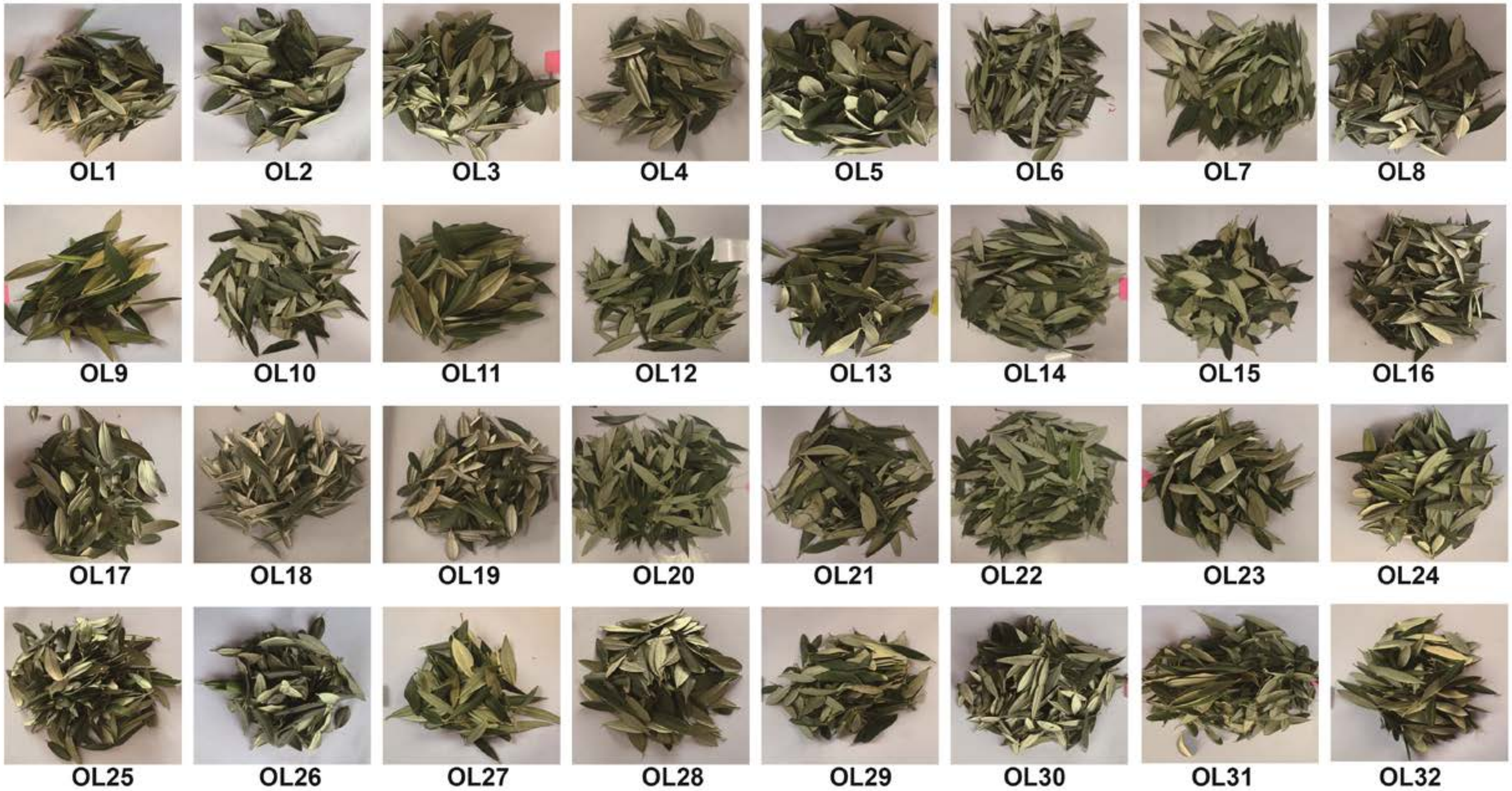

Figure S1. Leaves from the 32 investigated olive cultivars.

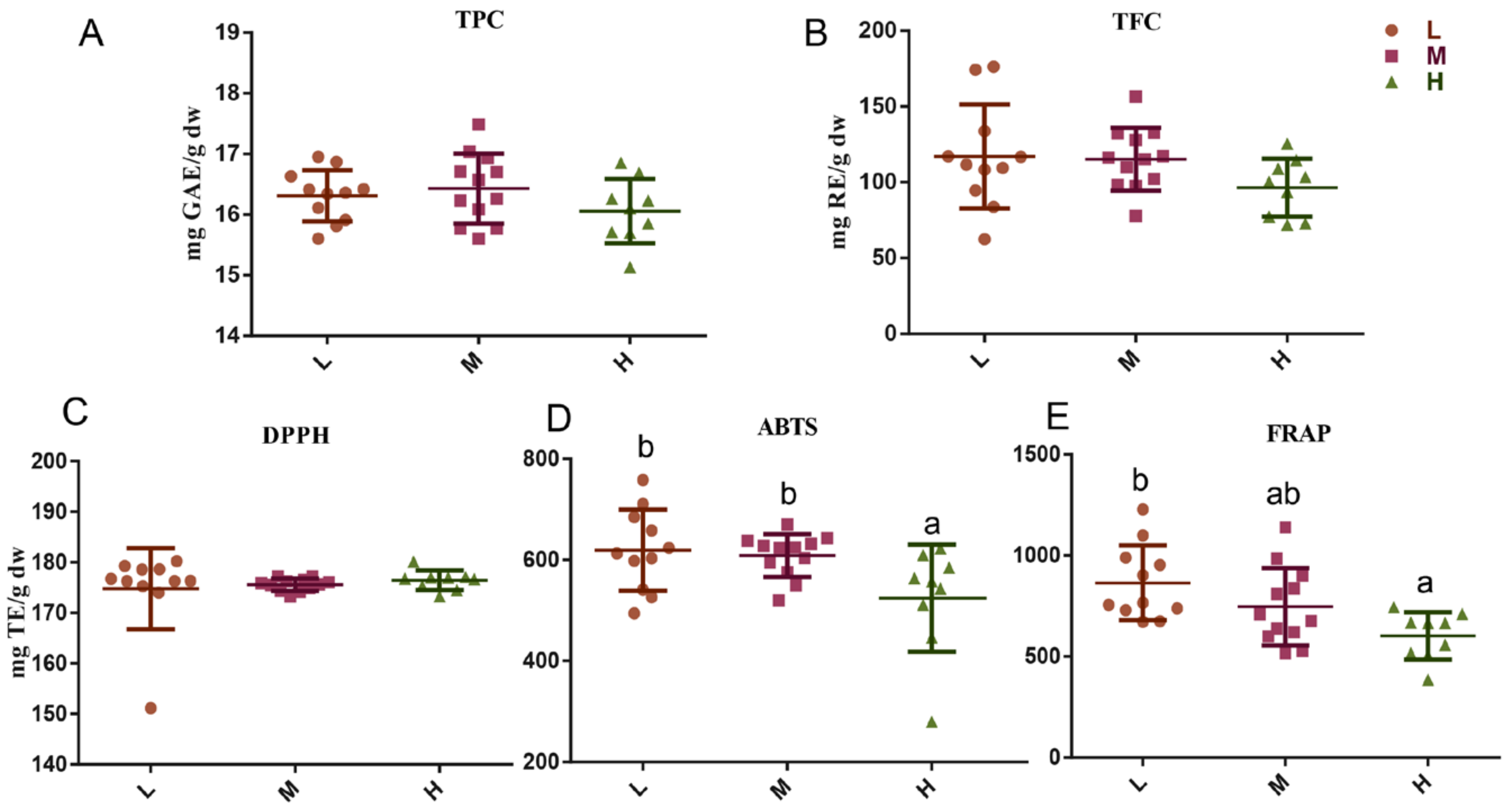

Figure S2. TPC, TFC, and antioxidant activities of olive leaf extracts from 32 cultivars.For each category, mean values followed by different letters indicate a significant difference ($P < 0.05$). H, M, and L represent high oil content (>20%) olive cultivars, medium oil content (16–20%) olive cultivars, and low oil content (<16%) olive cultivars, respectively.

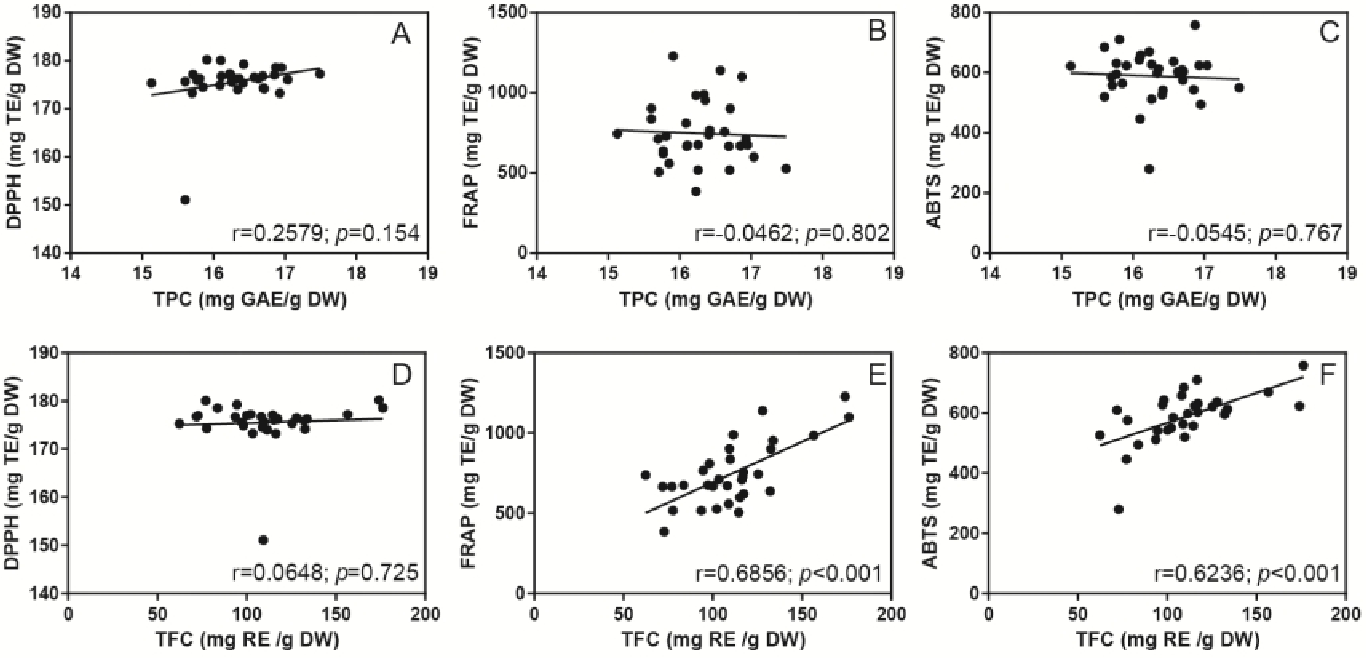

Figure S3. Pearson's correlation scatterplot of the relationships between (A) DPPH and TPC, (B) FRAP and TPC, (C) ABTS and TPC, (D) DPPH and TFC, (E) FRAP and TFC, and (F) ABTS and TFC.

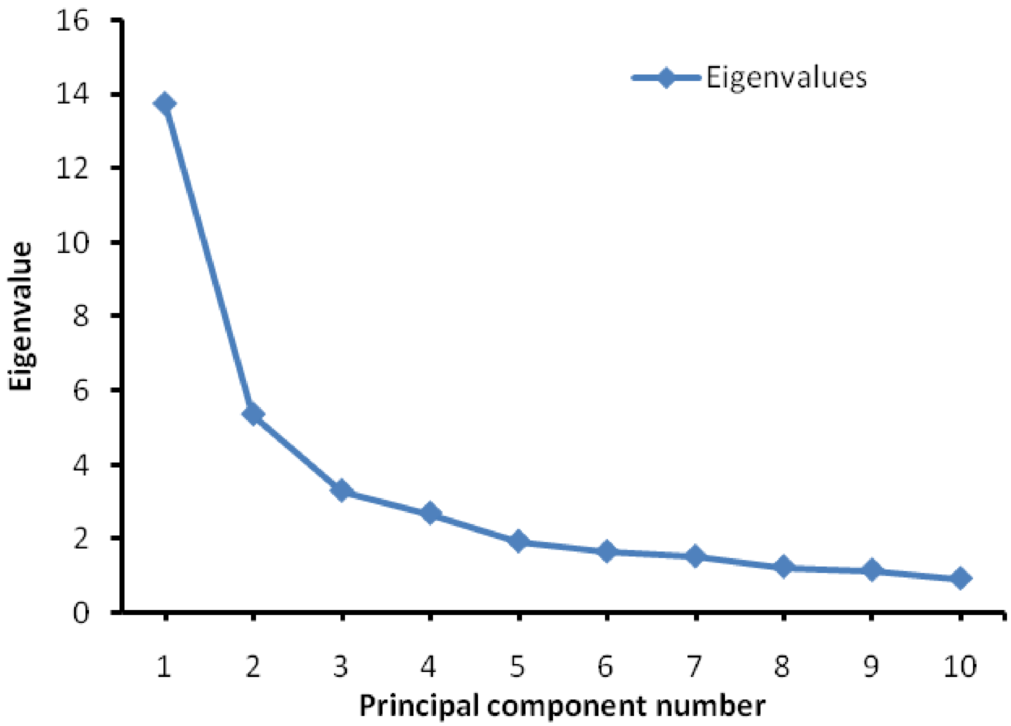

Figure S4. Eigenvalues for principal component analysis (PCA)
